# Agronomic Efficiency of Compost Extracts and Nitrogen-Fixing Bacteria in Soybean Crops

**DOI:** 10.3390/microorganisms13020341

**Published:** 2025-02-05

**Authors:** Andressa Pereira de Jesus, Mateus Neri Oliveira Reis, Lucas Loram Lourenço, Daniel José de Souza Mol, Layara Alexandre Bessa, Marivaine da Silva Brasil, Luciana Cristina Vitorino

**Affiliations:** 1 Laboratory of Agricultural Microbiology, Goiano Federal Institute (IFGoiano), Rio Verde Campus, Rio Verde 75901-970, GO, Brazil; andressamtv15@gmail.com; 2 Laboratory of Biodiversity Metabolism and Genetics, Goiano Federal Institute (IFGoiano), Rio Verde Campus, Rio Verde 75901-970, GO, Brazil; mateusnerioliveira@hotmail.com (M.N.O.R.); lucas.loram@outlook.com (L.L.L.); layara.bessa@ifgoiano.edu.br (L.A.B.); 3 SyncBio—Farm of Advanced Research for Regenerative Agriculture, GO-220 Road, Km 21, Montividiu 75915-000, GO, Brazil; djsmol2000@gmail.com; 4 Departamento de Ciências do Ambiente (DAM) (CPAN), Federal University of Mato Grosso do Sul (UFMS), Pantanal Campus, 1270 Rio Branco Ave., Corumbá 79304-902, MS, Brazil; marivaine.brasil@ufms.br

**Keywords:** regenerative agriculture, bioinputs, soil microbial biomass, leaf diseases, plant growth promotion

## Abstract

Regenerative agriculture and the use of bioinputs have been gaining prominence in the global agribusiness sector, driven by the growing demand for healthier foods produced with minimal impact on ecosystems. In this context, compost and its derivatives (compost extracts and teas) are used to provide effective microorganisms to crops, although production processes affect the efficiency of compost extracts, as well as the soil microbiota. Thus, the hypothesis raised was that the organic matter source used for compost formation affects the agronomic efficiency of compost extracts. The objective of this study was to evaluate the effect of compost extracts based on litterfall of angiosperm (AC) and gymnosperm (GC) species, and the use of inoculation with the nitrogen-fixing bacteria *Bradyrhizobium japonicum* and *Azospirillum brasilense* (*Bra+Azo*), on soil quality, crop growth, grain yield, and disease control in soybean (*Glycine max* L.) crops. Using AC and GC resulted in varying effects on soybean growth and soil microbial biomass carbon (SMBC), confirming the hypothesis that the organic matter source affects the agronomic efficiency of compost extracts. Plants inoculated with *Bra+Azo* exhibited higher chlorophyll contents, resulting in a higher photochemical yield than for those treated with compost extracts (AC and GC). However, plants inoculated with AC and GC exhibited high plasticity in mitigating photochemical stress, reaching similar photosynthetic and transpiration rates to those observed in plants inoculated with *Bra+Azo*. Additionally, inoculation with *Bra+Azo*, overall, improved the photosynthetic efficiency of soybean plants, and the compost extracts (AC and GC) were more effective than the inoculation with *Bra+Azo* in increasing soybean 1000-grain weight, probably due to improvements in root development. The growth promotion observed with AC and GC is likely attributed to increases in SMBC by these compounds, denoting improvements in soil quality and biocontrol of damage caused by insect attacks.

## 1. Introduction

Considering the need to meet the increasing world demand for foods, modern agriculture demands not only the development of new techniques and equipment that improve the management and increase the yield of crops, but also sustainable alternatives that address the high demand for chemical fertilizers and pesticides [1]. New agricultural practices have focused on reducing impacts on ecosystems, protecting non-target organisms, and recovering biodiversity in agricultural soils [2,3].

Therefore, regenerative agriculture and bioinputs have been used not only to ensure food production, but also to meet the increasing demand for healthier foods produced with low impact to ecosystems [4]. A reduction in the use of pesticides in food crops is expected by the adoption of these management practices, but this has been a major challenge [5,6].

The regenerative agriculture approach includes low-cost techniques that allow for the acquisition of effective microorganisms (EMs) [7]. Studies have shown that improvement in the diversity and dynamics of soil microbial communities results in cooperative aspects, boosting agricultural yields [8]. EMs interact symbiotically with their hosts and perform important functions; by producing toxins, they can protect host plants from colonization by phytopathogens and attacks from insects and herbivore mammals [9]. The use of compost extracts is among low-cost regenerative techniques, as it enables the obtainment of EMs that accelerate the natural decomposition of organic matter and promote the equilibrium of soil microbial flora, contributing to plant development [10,11]. On the other hand, the effectiveness of these low-cost techniques is often questioned by biological companies that produce microorganisms cultivated and formulated under aseptic conditions. In this context, the agricultural consumer market remains indecisive regarding the choice between techniques, which are frequently used in combination, such as seed co-inoculation with EMs and commercial strains. This scenario highlights the need for further discussion on the potential symbiotic or antagonistic interactions between the microorganisms involved.

The beneficial effects of compost extracts are explained not only by improving soil microbial flora, but also by providing nutrients and improving soil structure [12], contributing to the maintenance of soil fertility [13]. Furthermore, these compounds promote plant establishment and improve root development [14]. Despite being a well-established agricultural practice, the use of compost extracts and their derivatives (compost extracts and teas) in large-scale crops, such as soybean, maize, and cotton, remains relatively unexplored, because this technique was initially developed for family farmers [15]. However, the potential of compost extracts and their derivatives varies depending on the quality of production processes and the final product [16]. Thus, the hypothesis raised in the present study was that the organic matter source used to create composts affects the agronomic efficiency of compost extracts. In this sense, the effect of using leaves from angiosperm and gymnosperm species to develop composts was evaluated, because new-generation sequencing techniques have shown that different plant groups can shelter specific microorganisms [17] that contribute differently to the diversity of EMs in composts.

This hypothesis was tested by evaluating plant physiology, growth, and phytosanitary characteristics, and soil quality indicators. OJIP transient analysis is useful for assessing the effects of treatments on electron transport efficiency, quantum yields, and PSII structure and function [18]. However, soil microbial biomass carbon is an indicator of regenerative processes and efficiency in soil organic matter decomposition [19,20,21]. Additionally, disease control in crops is a major challenge in agriculture, and ensuring its effectiveness when using sustainable and regenerative methods is an even greater challenge.

Therefore, the importance of controlling leaf and soil diseases that challenge soybean production worldwide has been widely discussed. This discussion includes nematodes, phytopathogenic fungi, bacteria, viruses, and grazing and sucking insects that damage leaves, affecting photosynthesis and crop yields [22]. Economic losses can reach 100% yield loss when phytosanitary management is inappropriate [23,24].

The total estimated economic loss due to diseases for soybean crops in the USA, from 1996 to 2016, was US$ 95.48 billion [25]. The most common pathogens in soybean crops include *Microsphaera diffus*, *Cercospora kikuchii*, *Septoria glycines*, *Phakopsora pachyrhizi*, *Corynespora cassiicola*, *Erysiphe diffuse*, and others that attack seeds and roots [26]. Studies have shown that EMs can reduce or eliminate phytopathogens and insects, decreasing the incidence of diseases [27,28]. Thus, the objective of this study was to evaluate the effect of microorganisms present in the microbiota of compost extracts based on litterfall of angiosperm (AC) and gymnosperm (GC) species, as well as the use of inoculation with the nitrogen-fixing bacteria *Bradyrhizobium japonicum* and *Azospirillum brasilense* (*Bra+Azo*), on soil quality, crop growth, grain yield, and disease control in soybean (*Glycine max* L.) crops. This study aims to promote the use of compost extracts obtained using the Soil Food Web approach in large-scale crops.

## 2. Materials and Methods

### 2.1. Preparation of Compost Extracts

**Figure 1:**
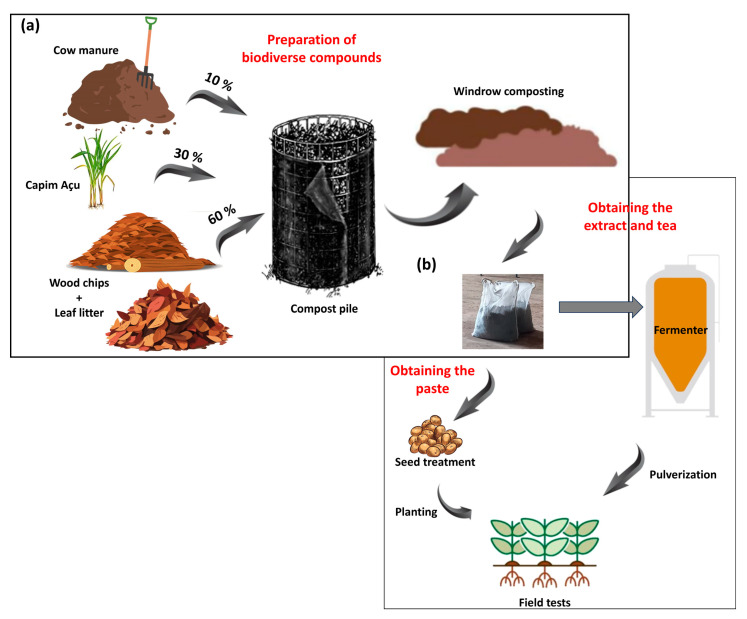
Preparation of compost extracts based on litterfall of angiosperm and gymnosperm species. Mixture of bovine manure, plant biomass (*Andropogon minarum*), and wood shavings, plus litterfall used to create compost piles and then arrange them in windrows (**a**). Use of compost for obtaining compost pastes and teas used for seed treatments and spray applications to crops, respectively (**b**).

Two compost extracts were prepared independently using different woody substrates: litterfall composed of leaves and branches of an angiosperm species (*Handroanthus impetiginosus*) (AC) and a gymnosperm species (*Pinus elliptti*) (GC). These compounds were obtained following the methodology proposed by Ingham [29] and Ingham and Slaughter [30], based on the Soil Food Web approach. The woody substrates accounted for 60% of the compost, which were enriched with 30% fresh plant biomass and 10% nitrogen-rich biomass. The woody substrates consisted of wood shavings plus litterfall (angiosperm or gymnosperm); the fresh plant biomass consisted of *Andropogon minarum* grass; and the nitrogen-rich biomass consisted of bovine manure (Figure 1a). The inputs used to prepare the two composts were similar for both woody substrates.

A total volume of 1200 L of each compost was obtained. The different inputs were homogenized and moistened to reach 50% moisture, as determined by Scheu et al. [31]. The homogeneous mixtures were placed separately in cylindrical piles covered with a 25 mm mesh screen, resulting in two piles, 1.5 m in height and 3 m in diameter. The mixture underwent a 30-day composting process, consisting of mesophilic, thermophilic, and maturation stages. Temperature and moisture content were monitored daily throughout the process, using a compost thermometer and the Soil Food Web approach, respectively [31]. The temperature was maintained at 35 ± 2 °C and the moisture at 40–45%. The compost piles were turned to cool the mixture, and moistened to increase moisture contents when needed.

They were arranged on the ground at the maturation stage, forming windrows; their moisture contents were monitored daily and maintained at 50%. The composts were enriched by adding 1 L of alga extract from the species *Ascophyllum nodosum* (Stingray^®^; Koppert, Piracicaba, Brazil) to each windrow. The composts were prepared to be used in a paste for seed treatment, and as extracts and teas for foliar spray applications (Figure 1b).

The compost extract paste was an adaptation of the Soil Food Web approach to increase the contact surface area between the compost microbiota and the seeds. Thus, the paste was produced by sieving 300 g of each compost extract twice in a 10 µ mesh, and adding 240 mL of water and 12 mL of alga extract to the sieved material.

The extract was obtained by washing 60 g L^−1^ of compost. The preparation was made in 1100 L polypropylene fermenters under constant air circulation. The compost was stored in bags made of a 400 µ mesh, which remained under water for 30 min, allowing for the extraction of microorganisms and organic components in the extracts. Then, the compost was filtered in bags made of 600 µ mesh, to avoid clogging of spraying nozzles. The compost tea was produced by fermenting this extract for 24 h and adding 0.130 mL L^−1^ of alga extract to the fermentation tank.

### 2.2. Study Areas and Sampling

The collection of litterfall used to create the composts and the field experiment were conducted at Brasilanda Farm in Montividiu, Goiás, Brazil (17°29′10″ S, 51°17′18″ W, and altitude of 905 m). The experiment was conducted during the 2022–2023 crop season, between 4 October 2022 and 8 February 2023. Climate data during the experiment were monitored by a meteorological station installed at the farm, using the FieldClimate software (https://metos.global/en/fieldclimate/, accessed on 7 April 2023) (Figures S1 and S2). Soil nutritional conditions for planting were assessed before the experiment’s implementation by evaluating three randomly collected soil samples from the experimental area (Table S1).

### 2.3. Seed Treatment, Planting, and Crop Management

The effects of the compost extracts were tested on soybean seeds of the cultivar DM-73I75, which has a medium cycle, maturity group 7.3, and resistance to cyst nematode. Six treatments were applied: AC compost extracts (01); GC compost extracts (02); inoculants based on *Bradyrhizobium japonicum* and *Azospirillum brasilense* (*Bra+Azo*) (03); AC compost extracts and *Bra+Azo* (04); GC compost extracts and *Bra+Azo* (05); and a control, using non-inoculated seeds (06). *B. japonicum* and *A. brasilense* inoculants were applied to the seeds using commercial products (Biomax^®^ Premium and Biomax^®^ Azum; Vittia, São Joaquim da Barra, Brazil), at rates recommended by the manufacturer (2.4 and 2.0 mL kg^−1^, respectively). Seed treatments using AC and GC pastes were applied at the rate of 50 g kg^−1^ (Table 1).

The seeds were planted soon after treatment, using a 30-row planter (Momentum^®^; Valtra, Mogi das Cruzes, Brazil). The crop was grown in an experimental area of 540 m^2^ (90 m^2^ per treatment), with treatments positioned side by side in the area. Each treatment was applied in six 30 m rows, with spacing of 50 cm between rows, and a density of 16 seeds per meter.

Treatments with AC and GC included the seed treatment and an additional treatment with the compost extract and compost tea, following the assumptions of the Soil Food Web approach developed by Ingham [29]. The extract was applied at the V3 vegetative stage, and the compost tea was applied at the R1 reproduction stage, using a backpack sprayer.

Fertilizers were applied based on the soil analysis, to meet the crop’s nutritional demand. Foliar applications were performed at the V3 stage, using urea (40% N, 3 kg ha^−1^) and manganese (12.7%, 200 g ha^−1^); at the V6 stage, using 250 g ha^−1^ of silicon dioxide (84–97%) + aluminum oxide (3–7%) + iron oxide (0.3–1.5%), 2 kg ha^−1^ of boron (9.2%), 2 kg ha^−1^ of MAP (11% N 60% P), 200 mL ha^−1^ of molybdenum (125 g L^−1^) + phosphorus (12.5 g L^−1^) + potassium (12.5 g L^−1^) + total organic carbon (75 g L^−1^), and 150 mL ha^−1^ of copper (5.5%); and at the R5 stage, using 1 L ha^−1^ of potassium oxide (28%) + manganese (3%) + S (13.6%) + boron (4%).

Control of pests and diseases was carried out using *Metarhizium anisopliae* and *Beauveria bassiana* (100 mL ha^−1^ at V2); the insecticide Cyptrin^®^ Prime (Tagros, Cuddalore, India) (150 mL ha^−1^ at V6 and R5.2); the insecticide Exalt^®^ (CTVA, Barueri, Brazil) (100 mL ha^−1^ at R5.4); the fungicide Unizeb^®^ Gold (UPL, Ituverava, Brazil) (1 L ha^−1^ at R5); and the insecticide Sperto^®^ (UPL, Ituverava, Brazil) (400 mL ha^−1^ at R5.4). Growth and physiological parameters were evaluated at the R5 stage (80 days from planting).

### 2.4. Physiological Evaluations

Plants were sampled in the two central rows of the evaluation area of each treatment. Each treatment was evaluated in 04 subplots, using five plants per subplot, totaling 20 experimental units per treatment. Leaves from the upper third of the plants were used for these evaluations.

Photosynthetic pigment indexes were analyzed using a portable chlorophyll meter (ClorofiLOG CFL1030; Falker^®^, Porto Alegre, Brazil) to determine chlorophyll *a*, *b*, and total levels. Pigment concentrations were given in Falker Chlorophyll Index (FCI).

Chlorophyll *a* fluorescence (OJIP transient) was determined using a portable fluorometer (FluorPen FP 100; Photon Systems Instruments, Drasov, Czech Republic). Leaves were dark-adapted for 30 min for full oxidation of the photosynthetic electron transport system. They were then subjected to a 3000 µmol m^−2^ s^−1^ blue light pulse, and measured for minimum fluorescence (F_0_) at 50 μs when all PSII reaction centers were open, known as the O phase, followed by J and I phases (at 2 and 30 ms, respectively), and measured for maximum fluorescence (F_M_) when all PSII reaction centers were closed, known as the P phase.

These values were used to estimate PSII bioenergetic indexes, as described by Strasser et al. [32]. The estimated parameters were as follows: specific light absorption flux per reaction center; energy flux captured per reaction center at *t* = 0; electron transport flux per reaction center; specific energy dissipation flux at the level of chlorophyll in the antenna complex; photosynthetic performance index, which incorporates energy cascade processes from the first absorption events to the reduction of PQ; maximum quantum yield of primary photochemistry; probability of an exciton moving an electron through the electron transport chain after quinone; and quantum yield of electron transport.

Gas exchanges were assessed using an infrared gas analyzer ( Li-6800; Licor, Lincoln, NE, USA). Plants were analyzed between 09:00 h and 10:00 h, using photosynthetically active radiation at 1000 μmol photons m^−2^ s^−1^, a block temperature of 27 °C, and a relative humidity of approximately 70%. The parameters measured were as follows: net photosynthetic rate (*A*) (µmol of CO_2_ m^−2^ s^−1^); stomatal conductance (mol of H_2_O m^−2^ s^−1^); transpiration rate (*E*) (mmol of H_2_O m^−2^ s^−1^); internal carbon concentration (*Ci*) (mmol m^−2^ s^−1^); carboxylation efficiency (*A*/*Ci*); and water use efficiency (*A*/*E*).

### 2.5. Phytosanitary Evaluations

Symptoms of diseases in the soybean plants were evaluated at the R 5.4 phenological stage. Plants were sampled in the two central rows of the evaluation area of each treatment, in the 04 subplots, using five plants per subplot.

A significant number of diseases can attack soybean plants during the crop cycle; thus, a scale was created to evaluate general symptoms without identifying specific diseases or pathogens. In this sense, the number of symptomatic leaves on each plant was assessed. The symptoms considered were those caused by grazing insects, such as caterpillars and beetles (perforations and cutouts in leaves), and those caused by phytopathogenic fungi (white, black, or yellowish spots and necrosis on the leaves).

### 2.6. Determination of Soil Microbial Biomass Carbon (SMBC)

SMBC was determined by the fumigation and extraction method [33]. Six samples of soil adhered to the plant roots within the two central rows of the evaluation area of each treatment were collected 95 days after planting. The analysis was conducted with 3 replications of fumigated samples and 3 replications of non-fumigated samples. The samples were stored in sterile Falcon tubes, and 20 g subsamples were taken and analyzed in triplicate, totaling 18 subsamples per treatment.

The subsamples were placed in glass flasks with lids, and 1 mL of chloroform (CHCl_3_) was added. These flasks were stored in the dark for 24 h, and then placed in a fume hood, and the lids were removed to allow for complete evaporation of chloroform; 50 mL of a K_2_SO_4_ (0.5 mol L^−1^) solution was then added, and the pH was adjusted to 6.7 ± 0.1. Subsequently, the samples were shaken for 30 min, and left to rest for 30 min, to allow the solid part to settle. The extract was obtained by removing the supernatant through filtration with filter paper (28 μm).

The microbial carbon in fumigated and non-fumigated extracts was determined using 8 mL of extract, with addition of 2 mL of a K_2_Cr_2_O_7_ (0.006 mol L^−1^) solution, 10 mL of H_2_SO_4_, and 5 mL of H_3_PO_4_. The mixture was cooled, 70 mL of distilled water was added, and then this mixture was cooled again. Four drops of diphenylamine (C_6_H_5_)_2_NH) (1 g in 100 mL of concentrated H_2_SO_4_) were then added, making the mixture change color from yellow to violet. Titration was performed in a 50 mL burette using an ammonium iron sulfate solution (Fe(NH_4_)_2_ (SO_4_)_2_ 6H_2_O) (0.033 mol L^−1^), with the samples kept under constant shaking until the color changed from violet to green. Flasks without soil were used as controls, and subjected to the same treatment as the fumigated and non-fumigated soil samples.

SMBC values were corrected based on the dry weight of the soil. Soil moisture was determined using 20 g of soil from each sample, which was dried in a forced-air oven (393/2; Tecnal, Piracicaba, Brazil) at 70 °C for 48 h. The soil was then weighed to obtain the dry weight.

SMBC was quantified using the following equation:

(1) $$\mathrm{FC}=\mathrm{difference}\;\mathrm{flux}\;\mathrm{obtained}\;\mathrm{by}:\;C\;(\mathrm{mg}\;\mathrm{kg}{-}^{1})=\frac{(Vb-Va)\times M\times 0.003\times V1\times 10^{6}}{DW\times V2}$$

where

Vb = volume of ammonium iron sulfate used in titration of control sample
Va = volume of ammonium iron sulfate used in titration of sample
M = exact molarity of ammonium iron sulfate
V1 = volume of extractor (K_2_SO_4_)
V2 = volume of extract
0.003 = carbon milliequivalent
DW = dry weight of soil
kc = correction factor (0.33), as described by Sparling and West (1988).

### 2.7. Evaluations of Agronomic and Yield Components

The agronomic parameters evaluated were as follows: shoot length (cm), shoot fresh weight (g), root fresh weight (g), nodule fresh weight (g), shoot dry weight (g), root dry weight (g), nodule dry weight (g), and number of nodules. The samples were weighed to obtain the fresh weight, and then dried in a forced- air oven (393/2; Tecnal, Piracicaba, Brazil) at 60 °C until constant weight to obtain their dry weights.

The yield components evaluated were the mean numbers of pods and grains, assessed in 10 plants per subplot, totaling 40 plants per treatment. Harvest was conducted using a John Deere S550 (John Deere, Horizontina, Brazil) combine harvester in 6 rows per treatment. Plots were harvested independently, and the harvester was cleaned and inspected between harvests of different treatments to prevent seed mixing. Borders were disregarded, and seeds were sampled during the filling of the harvester’s grain tank. The 1000-grain weight was obtained using a 50-seed acrylic grain counter (Dicalan).

### 2.8. Statistical Analyses

The data obtained from the different treatments were subjected to analysis of variance by the F test at a 5% significance level. Statistical differences between the means were evaluated by Tukey’s test at a 5% significance level. Alternatively, Dunnett’s test was used to compare the effects of each treatment, specifically focusing on comparison with the control treatment.

Subsequently, variables showing significant differences were jointly evaluated in a correlation matrix, and connected by principal component analysis (PCA). Considering that these variables had different units of measurement, PCA was conducted using standardized data, to obtain a mean of 0 and a standard deviation of 1. The number of principal components was defined based on eigenvalues greater than 1.0 and a cumulative explained variance over 70%.

The similarity matrix was developed to estimate similarities among plants from different treatments. The similarity index was obtained using the Pearson’s correlation coefficient, with *r* values transformed into distance values using the equation *d* = (1 − *r*) × 100. A dendrogram was then developed using the unweighted pair group method with arithmetic mean (UPGMA), with the fit between the distance matrix and the dendrogram estimated by the cophenetic correlation coefficient [34]; this analysis was conducted using the DendroUPGMA software (http://genomes.urv.cat/UPGMA/, 6 November 2024) [35]. All the statistical procedures were carried out using R statistical software version 4.4.0 [36].

## 3. Results

**Figure 2:**
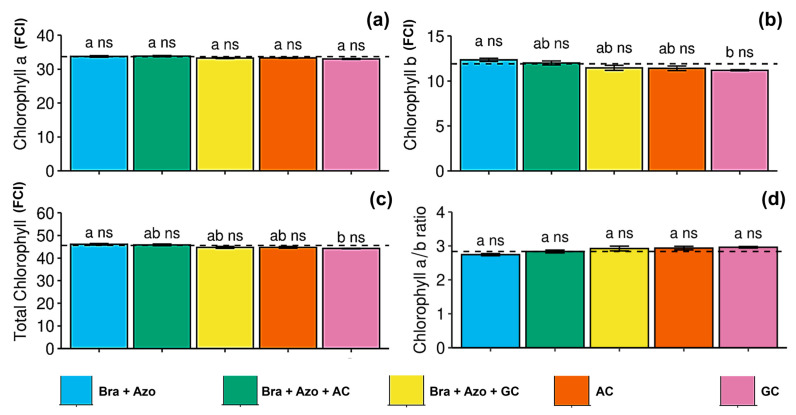
Photosynthetic pigments in soybean plants (*Glycine max* L.) under inoculation with compost extracts, combined or not with commercial strains of *Bradyrhizobium japonicum* (Bra) and *Azospirillum brasilense* (Azo). Data expressed in Falker Chlorophyll Index (FCI). Chlorophyll *a* (**a**), chlorophyll *b* (**b**), total chlorophyll (**c**), and chlorophyll *a/b* ratio (**d**). Composts produced based on litterfall of angiosperm (AC) and gymnosperm (GC) species. Dashed horizontal line represents mean of control treatment (non-inoculated plants). Bars with same letter are not significantly different from each other by Tukey’s test at 5% significance level; ns = not significantly different from control.

The treatments had no significant effect on leaf chlorophyll *a* content (Figure 2a), but plants inoculated with *Bra+Azo* tended to have a higher chlorophyll *b* content (12.34 ± 0.37 FCI) than those treated with GC (11.19 ± 0.14 FCI) (Figure 2b). However, these results did not significantly differ from those of the control (11.91 ± 0.12 FCI). Total chlorophyll contents were similarly affected, with plants inoculated with *Bra+Azo* (46.11 ± 0.84 FCI) presenting higher means than those treated with GC (44.23 ± 0.31 FCI), although the means were similar than those observed in the control treatment (45.58 ± 0.27 FCI) (Figure 2c). The chlorophyll *a/b* ratio was not significantly affected by the treatments with microbial inoculation (Figure 2d).

**Figure 3:**
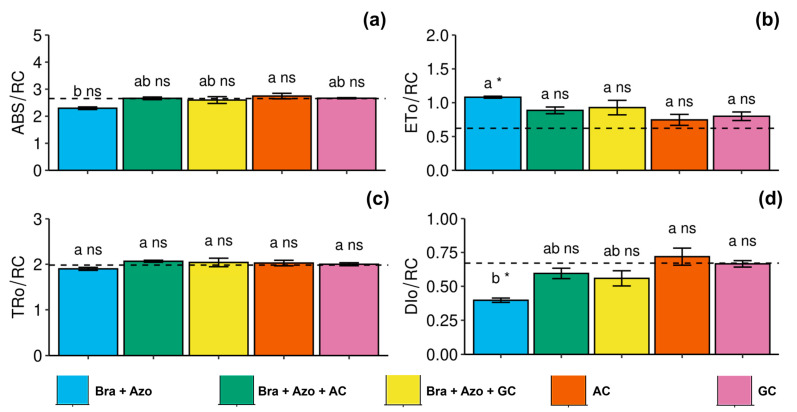
Primary photochemical indexes (chlorophyll *a* fluorescence) in soybean plants (*Glycine max* L.) under inoculation with compost extracts, combined or not with commercial strains of *Bradyrhizobium japonicum* (Bra) and *Azospirillum brasilense* (Azo). Specific light absorption flux per reaction center (ABS/RC) (**a**), electron transport flux per reaction center (ETo/RC) (**b**), energy flux captured per reaction center at *t* = 0 (TRo/RC) (**c**), and specific energy dissipation flux at level of chlorophyll in antenna complex (DIo/RC) (**d**). Composts produced based on litterfall of angiosperm (AC) and gymnosperm (GC) species. Dashed horizontal line represents mean of control treatment (non-inoculated plants). Bars with same letter are not significantly different from each other by Tukey’s test at 5% significance level; * and ns = significantly different and not significantly different from control, respectively.

The plants inoculated with AC presented high specific light absorption flux per reaction center (2.75 ± 0.22) in leaves, indicating a trend of photochemical stress. The values were higher than those of plants inoculated with *Bra+Azo* (2.29 ± 0.10) (Figure 3a). Electron transport flux per reaction center was higher in plants inoculated with *Bra+Azo* (1.08 ± 0.03) compared to the control plants (0.67 ± 0.05) (Figure 3b). Energy flux captured per reaction center at *t* = 0 did not differ among treatments, or between treatments and the control (Figure 3c). Similarly to specific light absorption flux per reaction center, energy dissipation as heat (specific energy dissipation flux at the level of chlorophyll in the antenna complex) also indicated photochemical stress in plants inoculated with AC and GC (0.71 ± 0.14 and 0.66 ± 0.05, respectively), and was low in plants inoculated with *Bra+Azo* (0.38 ± 0.03) (Figure 3d).

**Figure 4:**
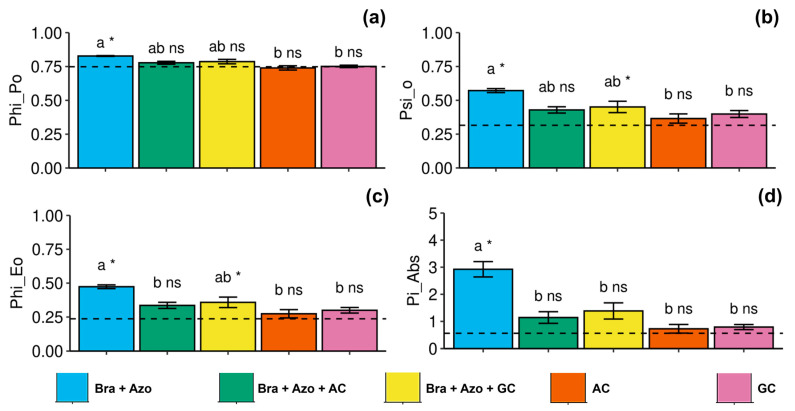
Primary photochemical indexes (chlorophyll *a* fluorescence) in soybean plants (*Glycine max* L.) under inoculation with compost extracts, combined or not with commercial strains of *Bradyrhizobium japonicum* (Bra) and *Azospirillum brasilense* (Azo). Maximum quantum yield of primary photochemistry (PHI_Po) (**a**), probability of exciton moving electron through electron transport chain after quinone (PSI_O) (**b**), quantum yield of electron transport (PHI_Eo) (**c**), and photosynthetic performance index (Pi_Abs) (**d**). Composts produced based on litterfall of angiosperm (AC) and gymnosperm (GC) species. Dashed horizontal line represents mean of control treatment (non-inoculated plants). Bars with same letter are not significantly different from each other by Tukey’s test at 5% significance level; * and ns = significantly different and not significantly different from control, respectively.

The maximum quantum yield of primary photochemistry was more effective in plants inoculated with *Bra+Azo* (0.83 ± 0.01), with higher means than the control plants (0.75 ± 0.06), and was negatively affected in plants inoculated with AC and AG (0.74 ± 0.03 and 0.75 ± 0.02, respectively) (Figure 4a). The probability of an exciton moving an electron through the electron transport chain after quinone was higher in plants inoculated with *Bra+Azo* (0.59 ± 0.22) and lower in plants inoculated with AC and GC (0.36 ± 0.07 and 0.39 ± 0.05, respectively). Control plants presented lower means than plants inoculated with *Bra+Azo* and *Bra+Azo*+GC (0.32 ± 0.02) (Figure 4b). Similarly to maximum quantum yield, the quantum yield of electron transport was higher in plants inoculated with *Bra+Azo* compared to those in the other treatments (0.48 ± 0.03), which presented higher means than the control plants (0.24 ± 0.05) (Figure 4c). The photosynthetic performance index was significantly higher in plants inoculated with *Bra+Azo* (2.86 ± 0.63) compared to those in other treatments and in the control (Figure 4d).

**Figure 5:**
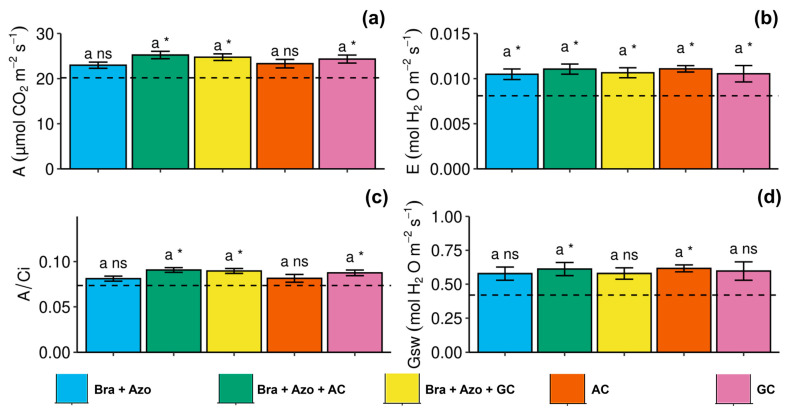
Gas exchanges in soybean plants (*Glycine max* L.) under inoculation with compost extracts, combined or not with commercial strains of *Bradyrhizobium japonicum* (Bra) and *Azospirillum brasilense* (Azo). Net photosynthesis (*A*) (**a**), transpiration rate (*E*) (**b**), carboxylation efficiency (*A/Ci*) (**c**), and stomatal conductance (*Gsw*) (**d**). Composts produced based on litterfall of angiosperm (AC) and gymnosperm (GC) species. Dashed horizontal line represents mean of control treatment (non-inoculated plants). Bars with same letter are not significantly different from each other by Tukey’s test at 5% significance level; * and ns = significantly different and not significantly different from control, respectively.

Gas exchanges were similar between treatments. However, the *Bra+Azo*+AC, *Bra+Azo*+GC, and GC treatments positively affected the net photosynthetic rate of soybean plants (25.22 ± 1.84, 24.75 ± 1.63, and 24.30 ± 2.10 µmol CO_2_ m^−2^s^−1^, respectively), resulting in higher rates than the control (20.40 µmol CO_2_ m^−2^s^−1^) (Figure 5a). Overall, plants with microbial inoculation showed higher transpiration rates than non-inoculated plants (0.007 ± 0.001 mol H_2_O m^−2^s^−1^) (Figure 5b). Similarly to net photosynthesis, carboxylation efficiency was higher in the *Bra+Azo*+AC, *Bra+Azo*+GC, and GC treatments than in the control (0.72 ± 0.006), with means of 0.86, 0.85, and 0.84, respectively (Figure 5c). The mean stomatal conductance in plants in the *Bra+Azo*+AC and AC treatments (both with 0.65 ± 0.05 mol H_2_O m^−2^s^−1^) was higher than that in the control (Figure 5d).

**Figure 6:**
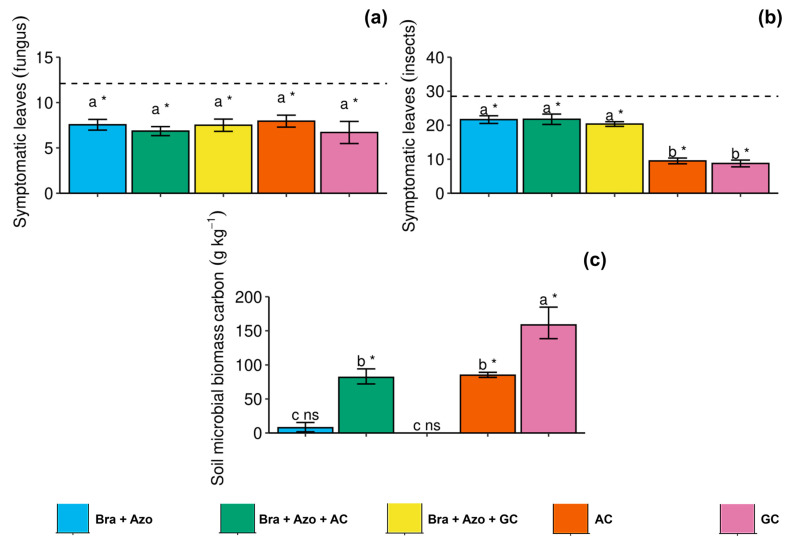
Incidence of disease symptoms in soybean plants (*Glycine max* L.) under inoculation with compost extracts, combined or not with commercial strains of *Bradyrhizobium japonicum* (Bra) and *Azospirillum brasilense* (Azo). Leaves attacked by fungi (**a**) or insects (**b**) and soil microbial biomass carbon (**c**). Composts produced based on litterfall of angiosperm (AC) and gymnosperm (GC) species. Dashed horizontal line represents mean of control treatment (non-inoculated plants). Bars with same letter are not significantly different from each other by Tukey’s test at 5% significance level; * and ns = significantly different and not significantly different from control, respectively.

The number of leaves with symptoms of diseases caused by phytopathogenic fungi did not differ between treatments with inoculation, but the treatments reduced the general incidence of symptoms of fungal diseases, with a significant effect compared to non-treated plants (12.10 ± 1.33) (Figure 6a). Similarly, microbial inoculation, overall, reduced the incidence of insect attacks. The AC and GC treatments considerably reduced insect attacks (9.50 ± 1.87 and 8.75 ± 2.22) compared to the other treatments (Figure 6b). The *Bra+Azo*+AC, AC, and GC treatments effectively increased the soil microbial biomass carbon (SMBC) (81.76 ± 21.56, 84.94 ± 7.25, and 158.61 ± 45.20 g kg^−1^ respectively) compared to the control (8.30 ± 1.22 g kg^−1^). However, inoculation with GC resulted in higher means than the other microbial treatments (Figure 6c).

**Figure 7:**
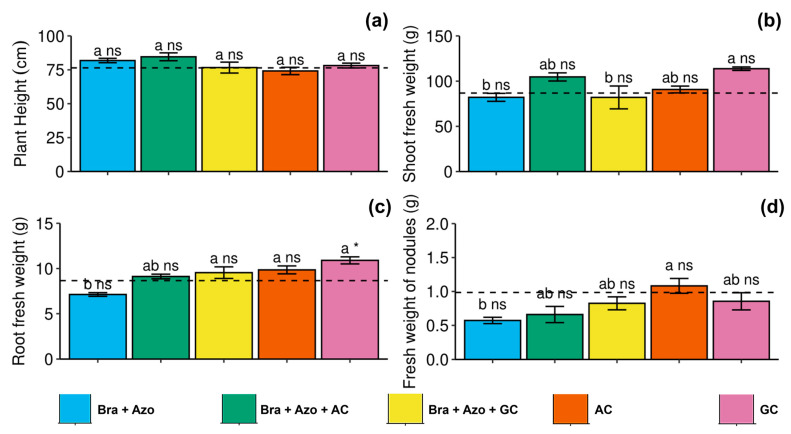
Biometric evaluations of soybean plants (*Glycine max* L.) under inoculation with compost extracts, combined or not with commercial strains of *Bradyrhizobium japonicum* (Bra) and *Azospirillum brasilense* (Azo). Plant height (**a**), shoot fresh weight (**b**), root fresh weight (**c**), and nodule fresh weight (**d**). Composts produced based on litterfall of angiosperm (AC) and gymnosperm (GC) species. Dashed horizontal line represents mean of control treatment (non-inoculated plants). Bars with same letter are not significantly different from each other by Tukey’s test at 5% probability level; * and ns = significantly different and not significantly different from control, respectively.

The microbial treatments had no significant effect on plant height (Figure 7a). Shoot fresh weight was higher in plants inoculated with GC (118 ± 4.35 g) compared to those inoculated with *Bra+Azo* (82 ± 9.87 g) and *Bra+Azo*+GC (81 ± 28.26 g) (Figure 7b). Root fresh weight was also higher in plants inoculated with GC (10.90 ± 0.87 g) compared to those inoculated with *Bra+Azo* (7.12 ± 0.45 g) and the control plants (8.66 ± 0.12 g) (Figure 7c). Nodule fresh weight was higher in plants inoculated with AC (1.08 ± 0.24 g), with higher means than those inoculated with *Bra+Azo* (0.57 ± 0.10 g) (Figure 7d).

**Figure 8:**
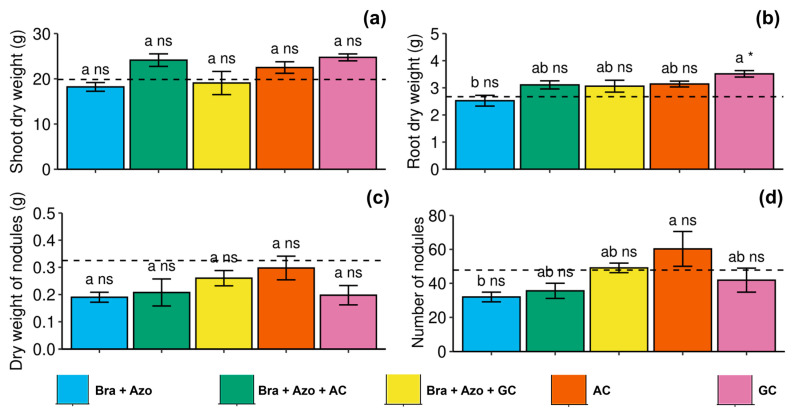
Biometric evaluations in soybean plants (*Glycine max* L.) under inoculation with compost extracts, combined or not with commercial strains of *Bradyrhizobium japonicum* (Bra) and *Azospirillum brasilense* (Azo). Shoot dry weight (**a**), root dry weight (**b**), nodule dry weight (**c**), and number of nodules (**d**). Composts produced based on litterfall of angiosperm (AC) and gymnosperm (GC) species. Dashed horizontal line represents mean of control treatment (non-inoculated plants). Bars with same letter are not significantly different from each other by Tukey’s test at 5% significance level; * and ns = significantly different and not significantly different from control, respectively.

Shoot and nodule dry weights were not significantly affected by the treatments (Figure 8a,c. Root dry weight was positively affected by inoculation with GC, presenting a higher mean (3.51 ± 0.24 g) than those found for plants inoculated with *Bra+Azo* (2.52 ± 0.13 g) and the control plants (2.67 ± 0.09 g) (Figure 8b). The number of nodules was higher in plants inoculated with AC (60.26 ± 22.94) and lower in plants inoculated with *Bra+Azo* (31.95 ± 6.34) (Figure 8d).

**Figure 9:**
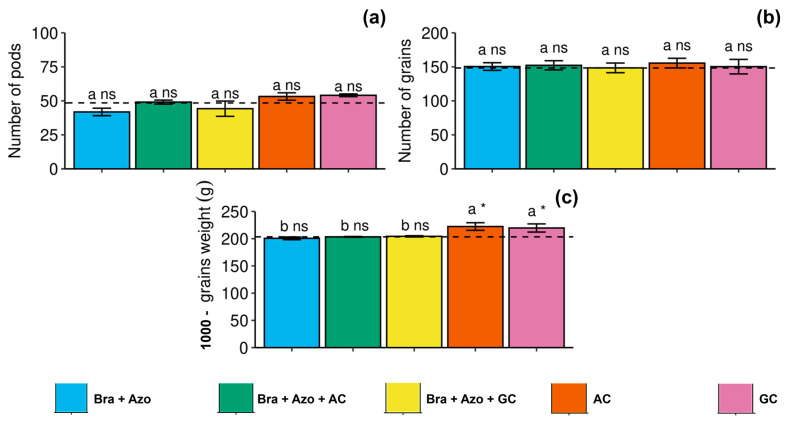
Yield components of soybean plants (*Glycine max* L.) under inoculation with compost extracts, combined or not with commercial strains of *Bradyrhizobium japonicum* (Bra) and *Azospirillum brasilense* (Azo). Number of pods (**a**), number of grains (**b**), and 1000-grain weight (**c**). Composts produced based on litterfall of angiosperm (AC) and gymnosperm (GC) species. Dashed horizontal line represents mean of control treatment (non-inoculated plants). Bars with same letter are not significantly different from each other by Tukey’s test at 5% significance level; * and ns = significantly different and not significantly different from control, respectively.

The number of pods and number of grains of soybean were not significantly affected by the treatments with microbial inoculation, presenting similar means to the control treatment (Figure 9a,b). However, 1000-grain weight was higher in plants inoculated with AC and GC (222.33 ± 5.50 and 219.66 ± 16.80 g, respectively) compared to the other treatments and the control (203.00 ± 6.22 g) (Figure 9c).

**Figure 10:**
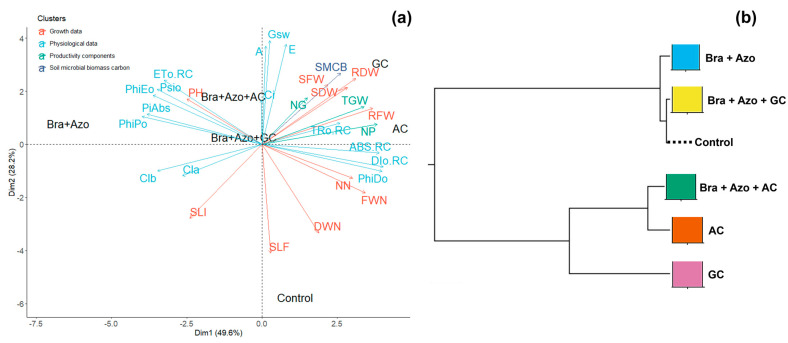
Principal component analysis for soil microbial biomass carbon and physiological, growth, and yield characteristics of soybean plants (*Glycine max* L.) under inoculation with compost extracts, combined or not with commercial strains of *Bradyrhizobium japonicum* (Bra) and *Azospirillum brasilense* (Azo) (**a**). Composts produced based on litterfall of angiosperm (AC) and gymnosperm (GC) species. Plant height (PH), shoot fresh weight (SFW), root fresh weight (RFW), nodule fresh weight (FWN), shoot dry weight (SDW), root dry weight (RDW), nodule dry weight (DWN), number of nodules (NN), number of pods (NP), number of grains (NG), 1000-grain weight (TGW), and leaves exhibiting symptoms of fungus attack (SLF) or insect attacks (SLI). Correlation cladogram between different microbial inoculation treatments and control treatment (**b**).

Principal component analysis (PCA) showed that the highest means for pigment production and photochemical performance explained the divergence observed between plants inoculated with *Bra+Azo* and plants in the other treatments. The highest number of leaves attacked by fungi or insects explained the variance in the control plants. Plants inoculated with GC and AC were connected to the lowest means of infestations. Although the worst photochemical performances were connected to the AC and GC treatments, these plants presented the highest fresh and dry weights of roots and shoots, number of pods, and 1000-grain weight. SMBC explained the differences observed between GC and the other inoculation treatments (Figure 10a). The correlation cladogram showed the formation of two treatment clusters. The AC and GC treatments were grouped together, indicating similar but slightly divergent effects on plants, as evidenced by the separation of GC. This separation was attributed to dissimilarities related to the SMBC data. However, the means of the control plants tended to be similar to those of plants inoculated with *Bra+Azo* and *Bra+Azo*+GC (Figure 10b).

## 4. Discussion

### 4.1. Soybean Plants (Glycine max L.) Inoculated with Bra+Azo Invest in Photosynthetic Pigments, Resulting in Higher Photochemical Yield than Plants Inoculated with Compost Extracts (AC and GC)

The inoculation of soybean seeds with *B. japonicum* and *A. brasilense* bacteria is well known for improving nitrogen biological fixation [37], resulting in significant economic and environmental benefits for soybean crops, as the N accumulated in the host plants is mainly transported through the xylem as ureides (allantoin and allantoic acid). Thus, the need for N can be met even in high-production cultivars [38,39]. N fixation rates in soybean plants can reach 372 kg ha^−1^ [40]. Studies have confirmed that strains of nitrogen-fixing bacteria can improve the production of photosynthetic pigments, indicating that chlorophyll content can be used as an indicator of the symbiotic efficiency of strains [41]. This occurs because most of the fixed N is directed towards the synthesis of photosynthesis-related enzymes, such as Rubisco, thus improving light use efficiency [42].

The inoculation with AC and GC compost extracts, which contain nutrients, organic matter, and microorganisms, combined with commercial strains, affected the activity of *B. japonicum* and *A. brasilense* bacteria and, consequently, the total chlorophyll content. This is attributed to the addition of nitrogen-rich biomass to the composts. Consequently, the addition of N to the system prevents bacteria from spending energy for N fixation, allowing them to use the supplied source. However, the establishment of commercial isolates in agriculture can be limited by competition with native microorganisms, indicating that the use of microbial combinations in agriculture should mitigate antagonism [43]. Wang et al. [44] suggest that while a high diversity of microbial combinations can promote stable and efficient plant growth, it may also increase susceptibility to the negative effects of microbial antagonism.

The reduced chlorophyll content in plants inoculated with AC and GC resulted in losses in photochemical performance, due to the smaller number of antenna complexes available to capture light. Antenna systems collect energy and conduct electrons to photosynthetic reaction centers through the electron transfer chain [45]. Therefore, the electron transport flux was higher in plants inoculated with *Bra+Azo*. According to Ort et al. [46], plants with higher chlorophyll content and more extensive antenna systems tend to absorb more light energy, which may provide a competitive advantage. However, under high-solar-radiation conditions, reducing leaf chlorophyll contents has been reported as a potential method to decrease excessive light absorption and improve photosynthetic solar energy conversion efficiency [46,47]. Although plants inoculated with AC and GC showed a high specific light absorption flux per reaction center and high energy dissipation as heat, they reached similar photosynthetic and transpiration rates to those inoculated with *Bra+Azo*, indicating a high ability to mitigate damage caused by excess light energy.

Reducing leaf chlorophyll content can improve light distribution in the canopy and increase light availability in the lower layers, thus improving the canopy photosynthesis [48,49]. This explains the higher photosynthetic and transpiration rates found for treatments with compost extracts compared to the control, despite their low photochemical performances.

Moreover, the application of compost tea directly to the leaf may have contributed to decreases in chlorophyll synthesis by directing the plant metabolism towards resistance responses to microbial colonization. Studies have shown that foliar applications using bacteria such as *Bacillus subtilis* and *B. amyloliquefaciens* can cause systemic resistance in cultivated plants [50,51], and can be used for managing diseases and pests [52]. Interactions between non-pathogenic or beneficial bacteria and plants mainly depend on plant growth regulators, such as salicylic acid, jasmonic acid, and ethylene, which are involved in the regulation of basal resistance against several pathogens [53]. Plant growth regulators are well known for inducing systemic resistance, which is similar to systemic acquired resistance (SAR), providing basal resistance to plants against pathogens and herbivores [54,55].

Overall, photosynthetic efficiency was higher in plants treated with microbial inoculation. Thus, the more effective biocontrol of fungi and insects in these plants, shown by the colonization of tissues by beneficial microorganisms and not by pathogenic microorganisms, resulted in greater use of light energy. This is explained by the presence of foliar pathogens, which resulted in stomatal closure, reducing stomatal conductance and consequently photosynthesis [56].

### 4.2. Compost Extracts Based on Litterfall of Angiosperm and Gymnosperm Species Are More Effective than Inoculation with Bra+Azo in Promoting Growth and Increasing Soybean 1000-Grain Weight

This result can be attributed to the action of efficient microorganisms (EMs), such as environmental probiotics, which promote crop growth and health through N fixation, K and P solubilization, release of trace elements, secretion of exopolysaccharides, transformation of organic matter into available nutrients, increased soil water retention capacity, and improved soil health [57]. They also release bioactive compounds, such as vitamins, hormones, and enzymes that stimulate plant growth [58]. Avila et al. [9] indicate that the positive effects of crop colonization by EMs are associated with their effects in accelerating natural decomposition of organic matter and improving soil microbial balance, promoting the regeneration of rhizosphere communities that affect crop growth and yield.

The use of a compost extract paste, rich in EMs, was an adaptation of the Soil Food Web approach for seed treatment to increase the contact surface between microorganisms and seeds. The paste’s composition was complemented with an *Ascophyllum nodosum* extract. This alga extract is known for improving soybean grain yield, especially when the plants are subjected to abiotic stress conditions [59,60].

Plants inoculated with AC and GC tended to invest more in the root system and nodules. This investment compensated for photochemical stress and lower chlorophyll content in the leaves. EMs can mineralize and provide essential nutrients to plants, activating metabolism and root growth [61]. According to Pugas et al. [62], EMs are added to the soil to increase microbiological diversity, promote organic matter decomposition, and consequently release nutrients more efficiently in agricultural soils. Microbial combinations are effective in providing nutrients for soybean crops [63].

The better root development of plants inoculated with AC and GC resulted in higher 1000-grain weights, because grain filling depends on nutrient uptake, which is used for the formation of important molecules, such as proteins, carbohydrates, and fatty acids [64]. Bajagić et al. [65] report that the combined application of EMs and NPK fertilizers can result in increases of 15.67% in soybean yield, 0.34% in protein content, and 0.47% in lipid content in soybean grains.

### 4.3. The Growth Promotion Provided by AC and GC Compost Extracts Is Explained by the Improvement in Soil Microbial Biomass Carbon (SMBC) and the Biocontrol of Insect Attacks

Compost extracts consisted of a potentially multifunctional microbiota with a high organic matter content, in which organic substances such as humic and fulvic acids and humin were biologically active. The synthesis of humic acids is facilitated by conditions of high microbial diversity, which boost the production of decomposition enzymes [66]. Although the mechanisms by which the chemical structure of these substances affects plant metabolism are still under debate [67], Meerza et al. [68] showed that the treatment of soybean plants with humic acids resulted in greater plant growth in crops grown in different periods.

SMBC is used as an indicator of soil quality [69]. Total microbial biomass represents a small portion of the soil; however, it is important because it is the most labile part of soil organic matter [70]. Moreover, the amount of nutrients stored in soil microbial biomass can reach up to 600 (N) and 300 (P) kg ha^−1^ in the top 30 cm of soil, sometimes exceeding the annual amount of nutrients applied as fertilizer [71]. The AC and GC compost extracts were effective soil conditioners for the soybean crops, as the microbial communities selected in the composting process regenerate the microbiota of agricultural soils [72], increasing the microbial abundance and diversity and the possibility of rhizosphere colonization with microorganisms that express important functional characteristics [73]. Several factors affect the establishment and colonization of soil by microbial communities, including soil texture, pH, nutrient content, moisture, and temperature, and competition among microorganisms [74]. Thus, the microorganisms selected in GC showed a better response to crop conditions. Oliveira et al. [75] reported that, similarly to compost extracts, carbon-rich organic compounds increase SMBC. These compounds can retain nutrients in the soil and keep them available for the biological activity of microbial communities.

However, the evaluated compost extracts were effective in reducing insect attacks on soybean leaves. This biocontrol is attributed to the production of a protective biofilm or antibiosis. According to Ingham [29], coating leaves with compost teas is an efficient method for preventing diseases in several crops. Compost teas contain aerobic microorganisms that form a protective layer on tissues, which is associated with proteins, extracellular carbohydrates, and exopolysaccharides [76]. The toxins released by this layer can repel insects [77]. Studies have shown that bacteria produce specialized metabolites that have strong larvicidal effects against flies, and larvae that ingest spores of streptomyces die. The mechanism of toxicity is specific to the chemistry of the bacterium: bacteria that produce cosmomycin D cause a response, with similar cell death to that occurring in the larval digestive tract; bacteria that produce avermectin cause paralysis [78]. 

*Bacillus* is one of the largest genera within the phylum Firmicutes, with biocontrol species reported in organic waste composts [79]. Studies have shown that Gram-positive bacteria consolidate during the thermophilic stage of composting, because they form endospores that become resistant to high composting temperatures. Some *Bacillus* species have beneficial effects on plants, producing siderophores, solubilizing nutrients, and preventing pathogen attack [80,81]. Additionally, actinobacteria are abundant in EM-treated composts. These bacteria can degrade organic matter and release antibiotics and bioactive compounds that promote growth and protect plants [82,83,84].

Therefore, the use of compost extracts produced using the Soil Food Web approach is recommended for regenerating the microbiota of agricultural soils with soybean crops, focusing on plant growth promotion and biocontrol of pests. The agronomic efficiency of compost extracts has been proven, opening up prospects for regenerative agriculture involving the application of composting methods to large-scale crops.

## 5. Conclusions

Soybean plants inoculated with *Bradyrhizobium japonicum* and *Azospirillum brasilense* produced more chlorophyll, resulting in a higher photochemical yield than those inoculated with compost extracts based on litterfall of angiosperm and gymnosperm species. However, plants inoculated with AC and GC showed a significant response to photochemical stress, reaching similar photosynthetic and transpiration rates to those observed in plants inoculated with *Bra+Azo*. Overall, microbial treatment improved the photosynthetic efficiency of soybean plants, and compost extracts (AC and GC) were more effective than *Bra+Azo* inoculation in increasing 1000-grain weight, which was probably a response to improvements in root development. The plant growth promotion achieved using AC and GC can be attributed to increases in soil microbial biomass carbon promoted by these compost extracts, denoting an improvement in soil quality and biocontrol of damage caused by insect attacks. The distinct effects of AC and GC on growth promotion and soil quality in soybean crops support the hypothesis that the source of organic matter influences the agronomic efficiency of compost extracts. However, we recommend the application of AC and GC in *G. max* cultivation, but not in combination with *Bra*+*Azo*.

## Figures and Tables

**Table 1:** Summary of inoculation treatments used to evaluate effects of compost extracts, alone or in combination with commercial strains of *Bradyrhizobium japonicum* (*Bra*) and *Azospirillum brasilense* (*Azo*), on growth and physiological characteristics of *Glycine max* L. DAA = days after application.

Code Treatment	Composition	Dose (Kg^−1^ of Seed)	Phenological Phase of Application
Control	Non-inoculated plants	-	-
AC	AC compost extracts (*Handroanthus impetiginosus*)	50 g	Seed, V3 (35 DAA), and R1 (55 DAA)
GC	GC compost extracts (*Pinus elliptti*)	50 g	Seed, V3 (35 DAA), and R1 (55 DAA)
*Bra+Azo*	*Bradyrhizobium japonicum* + *Azospirillum brasilense*	2.4 + 2.0 mL	Seed
*Bra+Azo* + AC	*Bradyrhizobium japonicum* + *Azospirillum brasilense* + AC compost extracts	2.4 + 2.0 mL + 50 g	Seed, V3 (35 DAA), and R1 (55 DAA)
*Bra+Azo* + GC	*Bradyrhizobium japonicum* + *Azospirillum brasilense* + GC compost extracts	2.4 + 2.0 mL + 50 g	Seed, V3 (35 DAA), and R1 (55 DAA)

## Data Availability

The original contributions presented in this study are included in the article/Supplementary Material. Further inquiries can be directed to the corresponding author.

## Supplementary Materials

The following supporting information can be downloaded at https://www.mdpi.com/article/10.3390/microorganisms13020341/s1: Figure S1: Temperature and solar radiation during the cycle of soybean plants (*Glycine max* L.) grown under the inoculation of compost extracts, combined or not with commercial strains of *Bradyrhizobium japonicum* (Bra) and *Azospirillum brasilense* (Azo). Composts produced based on litterfall of angiosperm (AC) and gymnosperm (GC) species.; Figure S2: Relative air humidity and rainfall depths during the cycle of soybean plants (*Glycine max* L.) grown under the inoculation of compost extracts, combined or not with commercial strains of *Bradyrhizobium japonicum* (Bra) and *Azospirillum brasilense* (Azo). Composts produced based on litterfall of angiosperm (AC) and gymnosperm (GC) species.; Table S1: Chemical and texture analyses of the soil of the experimental area used to evaluate the effect of inoculation of soybean plants (*Glycine max* L.) with compost extracts, combined or not with commercial strains of *Bradyrhizobium japonicum* (Bra) and *Azospirillum brasilense* (Azo). Composts produced based on litterfall of angiosperm (AC) and gymnosperm (GC) species.
