# Supplementary material for: Agronomic Efficiency of Compost Extracts and Nitrogen-Fixing Bacteria in Soybean Crops
Source: Microorganisms. 2025 Feb 5;13(2):341. doi: 10.3390/microorganisms13020341 (PMC11858144; doi:10.3390/microorganisms13020341)
Supplement: Supplementary file 1 [file microorganisms-13-00341-s001.zip › microorganisms-3450889-supplementary.pdf]

Supplementary material

Agronomic efficiency of compost extracts and nitrogen-fixing bacteria in soybean crops

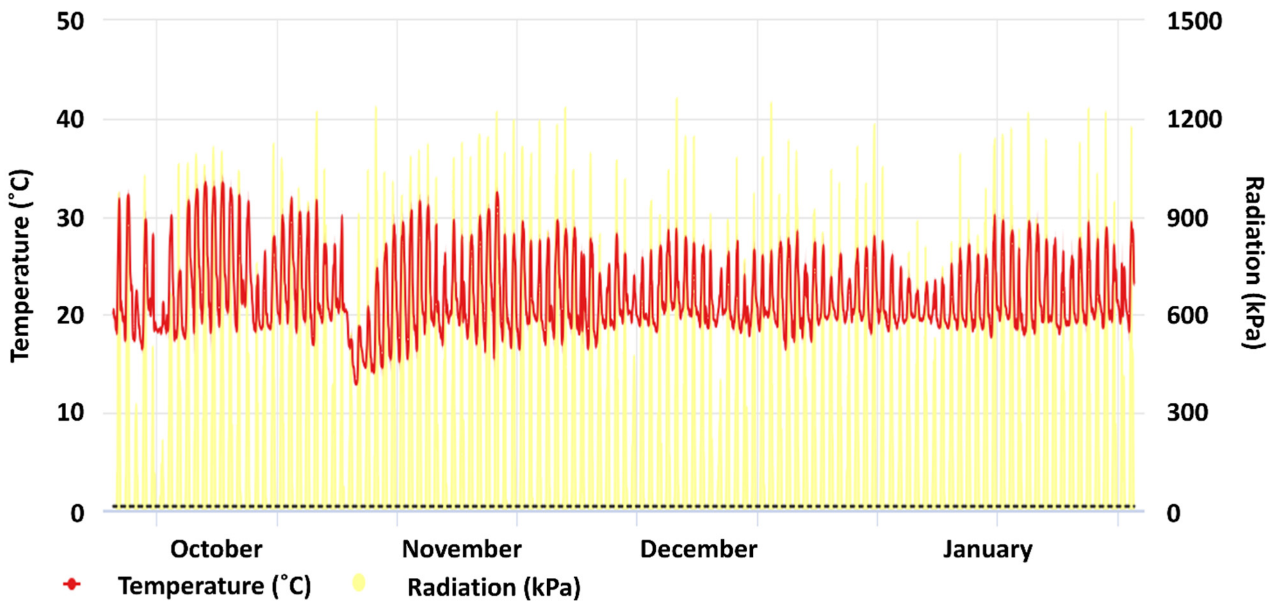

Figure S1. Temperature and solar radiation during the cycle of soybean plants (*Glycine max* L.) grown under the inoculation of compost extracts, combined or not with commercial strains of *Bradyrhizobium japonicum* (Bra) and *Azospirillum brasilense* (Azo). Composts produced based on litterfall of angiosperm (AC) and gymnosperm (GC) species.

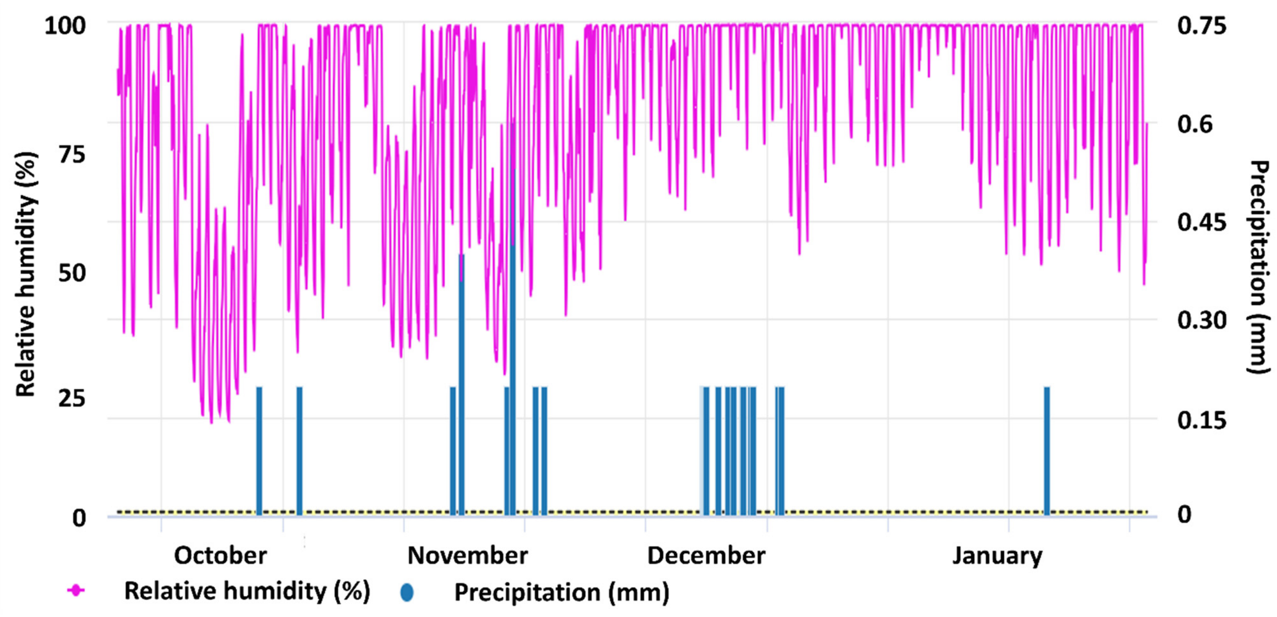

Figure S2. Relative air humidity and rainfall depths during the cycle of soybean plants (*Glycine max* L.) grown under the inoculation of compost extracts, combined or not with commercial strains of *Bradyrhizobium japonicum* (Bra) and *Azospirillum brasilense* (Azo). Composts produced based on litterfall of angiosperm (AC) and gymnosperm (GC) species.

Table S1. Chemical and texture analyses of the soil of the experimental area used to evaluate the effect of inoculation of soybean plants (*Glycine max* L.) with compost extracts, combined or not with commercial strains of *Bradyrhizobium japonicum* (Bra) and *Azospirillum brasilense* (Azo). Composts produced based on litterfall of angiosperm (AC) and gymnosperm (GC) species.

**Ca**	**Mg**	**Ca+Mg**	**Al**	**H+Al**	**K**	**K (total)**	**S**	**P**	**$CaCl_2$**
----------------- $cmol_c\ dm^{-3}$ -----------------						--------- $mg\ dm^{-3}$ ---------			pH
4.46	1.17	1.80	0.02	2.39	53.80	170.96	17.40	15.67	5.28

**Na**	**Fe**	**Mn**	**Cu**	**Zn**	**B**	**CEC**	**SB**	**BS**	**AS**
--------------- $mg\ dm^{-3}$ ---------------						$cmol_c\ dm^{-3}$	%	%	%
1.00	21.40	14.93	1.44	6.36	0.46	9.71	59.09	47	1.40

**Texture ($g\ kg^{-1}$)**			**OM**	**Ca/Mg**	**Ca/K**	**Mg/K**	**Ca/CEC**	**Mg/CEC**	**K/CEC**
Clay	Silt	Sand	$g\ dm^{-3}$	----------------- Ratio between bases -----------------					
550	80	400	26.83	1.10	2.90	2.70	45.67	12.01	1.41

P, K, Na, Cu, Fe, Mn, and Zn extracted by Mehlich 1; Ca, Mg, and Al extracted by KCl 1 mol L^{-1}; S extracted by Ca $(H_2PO_4)_2$ 0.01 mol L^{-1}; OM extracted by the colorimetric method; B extracted by hot water; CEC = cation exchange capacity; SB = sum of bases; BS = based saturation; AS = aluminum saturation; and OM = organic matter.
